# Restructuring the Production of Medicines: An Investigation on the Pharmaceutical Sector in China and the Role of Mergers and Acquisitions

**DOI:** 10.3390/ijerph14101179

**Published:** 2017-10-05

**Authors:** Elisa Barbieri, Manli Huang, Shenglei Pi, Mattia Tassinari

**Affiliations:** 1 Department of Economics and Statistical Sciences, University of Udine, 33100 Udine, Italy; elisa.barbieri@uniud.it; 2 School of Business Administration, South China University of Technology, Guangzhou 510640, China; 3 Guangzhou Academy of Social Science, Guangzhou 510410, China; pishenglei@126.com; 4 Department of Economics and Management, University of Ferrara, 44121 Ferrara, Italy; mattia.tassinari@unife.it

**Keywords:** China, mergers and acquisition, M&A, pharmaceutical, industry, composite indicators, industrial policies

## Abstract

In places like China, an ageing population coupled with changes in living standards and increases in disposable income, imply a shift of the demand for health-related goods and services which is likely to affect the whole organization of the industries that supply such goods and services at the global level. One of the industries most likely to be affected is the pharmaceutical sector. In the early 2000s China was already the second largest global producer of pharmaceutical ingredients. The pharmaceutical sector has become one of the most important industries promoted by the Chinese government and *Five-Year Plan of China’s Strategic Emerging Sectors*, mergers and acquisition (M&A) activity has been the key strategy to restructure the sector and increase its competitiveness. This paper firstly provides an updated picture of the evolution of M&As in the pharmaceutical sector, compared to other sectors, in China in the period 2005–2013. Secondly, we develop a composite indicator to measure the industrial performance of all Chinese industrial sectors over time, which allows us to assess the performance of the pharmaceutical industry compared to that of other sectors of the Chinese economy. Finally, we develop and estimate an empirical model that tests the relationship between the number of M&A in a sector and its performance, with a particular focus on the pharmaceutical case. The results offer some initial evidence of positive effects from the process of restructuring of the pharmaceutical sector in China.

## 1. Introduction

A few important recent mega-trends are affecting global demand for health-related goods and services. Among these is the ageing of population, which is of interest to most industrialized western nations, as well as to some big emerging countries, including China [1]. Population ageing together with changes in living standards and increases in disposable income in large economies like China imply—sooner or later—a shift in demand for health-related goods and services. These global trends, together with the centrality of the knowledge economy and research networks [2], place certain manufacturing sectors at the core of contemporary international industry. The pharmaceutical sector is without a doubt one of these cases [3,4,5]. This sector is already experiencing changes in the production strategies of both emerging countries and western industries which are likely to affect what medicines are produced and where: new countries are entering global value chains and are gradually becoming reference hubs for the realization of drugs or drug components [6,7]. In the future, this might have effects on the improvement of health in specific parts of the world, on the location of Research and Development (R&D) investment supporting such improvements and on the role that some manufacturing companies and some regions will play in the global market.

China is a particularly interesting case-study in this context. In the early 2000s the country had already become the second largest producer of pharmaceutical ingredients in the world [8]. The pharmaceutical sector has been one of the most rapidly emerging industries in China. For the last two decades, the sector has experienced double-digit annual growth rates, with an average of 16.1 percent annual growth in production value between 1978 and 2005 [9]. These growth rate further increased to 20 percent from 2006 to 2012 [10]. Meanwhile, the international competitiveness of China’s pharmaceutical sector has also been improving fast. Thanks to such rapid growth, the pharmaceutical sector has become one of the most important industries promoted by the Chinese governments. Starting from the 10th *Five-Year Plan for China National Economic and Social Development* and up to the latest 13th Plan, the pharmaceutical sector has been identified as one of the “strategic emerging sectors”. In particular, in the 12th *Five-Year Plan of China’s Strategic Emerging Sectors*, mergers and acquisitions (M&As) across firms in the pharmaceutical industry were already strongly encouraged. It emerges clearly from the related documents [11] that M&As have been the key strategy of the Chinese government to restructure the sector in this important phase. By allowing M&As, the government wishes to stimulate the creation of national champions able to compete at the global level in the supply of pharmaceutical products [12,13,14].

From this perspective, studying M&A in the pharmaceutical sector in China can provide unique insights on the future evolution of the global supply of medicines. In addition, from a more scholarly perspective, M&A in the pharmaceutical sector have been considered particularly interesting in the wider literature on industrial economics, for several reasons.

Firstly, compared to other industries, the pharmaceutical sector is particularly active in M&As at the world level and it provides a good laboratory to investigate the effect of M&As on the performance of the sector (i.e., ability to discover new medicines, engagement in new R&D investment and etc.) [15].

Secondly, the industry has important distinctive features, related in particular to the high sunk costs required to develop and sell new drugs—including R&D investment, trials and marketing – and to the high risks associated with its drug pipeline. For these reasons, the major findings on the success of M&As in terms of ex-post efficiency and profitability suggest that synergy-seeking M&As are particularly effective in the pharmaceutical sector [16,17,18].

Thirdly, the debate on the effectiveness of M&As in the pharmaceutical industry in the contemporary global economy is inconclusive [19]. On the one hand, previous studies emphasize that large pharmaceutical firms tend to gain higher returns after M&As than small and medium sized ones [20]. However, on the other hand, Berndt [21] argues that this positive association is contingent upon the timing of the operation: if the M&A takes place within the period of validity of the major patents of the target firm, then the positive association between size and profitability holds; however, in the long run this association tends to become negative. Finally, and particularly pertinent for our case, existing studies are mainly limited to the pharmaceutical sector in U.S. and Europe. Little is known about the evolution and the effectiveness of M&As in the pharmaceutical sector in China. In the Chinese academic literatures there are a few theoretical analyses on this topic [22], but very few empirical studies investigate the relationship between the restructuring of the pharmaceutical sector through M&As and the competitiveness of the industry (most studies deal with the effects of M&As at the firm level. For a review of the Chinese literature on this see Li [23]). As we shall see, the pharmaceutical sector in China, much more than other industries, is experiencing a process of restructuring through M&As. It is our purpose to investigate whether such restructuring is actually associated with above-average improvements in the performance of the sector and under what conditions.

This paper seeks to contribute to the extant literature in several ways. First of all we provide an updated picture of the evolution of M&As in the pharmaceutical sector in China compared to other sectors, over the period 2005–2013. In doing so, we distinguish the type of M&A (horizontal, vertical and conglomerate), the origin of the target and acquirer firms and then focus, in particular, on the M&As undertaken between Chinese firms. Secondly, we develop a composite indicator to measure the industrial performance of all Chinese industrial sectors over time, which allows us to assess the performance of the pharmaceutical industry compared to that of other sectors of the Chinese economy. Finally, we develop and estimate an empirical model that tests the relationship between the number of different types of M&A in a sector and its performance, with a special focus on the pharmaceutical case.

## 2. Recent Evolutions in the Pharmaceutical Sector in China

**Figure 1:**
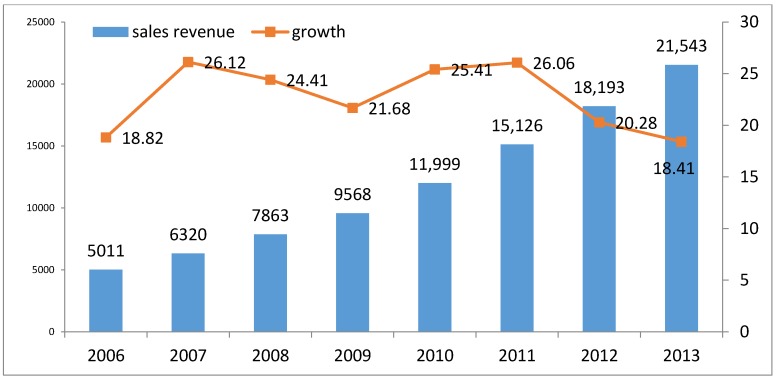
Sales revenue and its growth in pharmaceutical sector. Source: WIND database (2016).

Pharmaceuticals represent a major fast-growing sector in China. Sales revenues in the Chinese pharmaceutical sector have been continuously improving in the period 2005–2013, with an annual growth rate of between 20% and 26% (see Figure 1). During China’s Tenth Five-year Plan (2001–2005), the ratio of profits to sales in the sector was below 9%, but since 2007 it has constantly increased. In 2010 it reached a peak of 11.67% and remained above 10% in 2012 and 2013 [4]. In terms of total production output, the Chinese pharmaceutical sector shifted from 2% of world production in 2002 to 6% in 2011 [4].

In the post-crisis period the sector has undertaken a particularly significant process of restructuring through M&As. Although, as highlighted by other authors [24], M&As were not new in the sector, it can be argued that after 2009 the pace of the restructuring process has notably changed. The main actors of this new wave of M&As have been Chinese listed companies. Given the generally large size of such companies, M&A are characterized by an increasing average transaction value of each operation, especially after 2009. Data from our database (see Section 4) show that, in terms of number of operations, M&A in the Chinese pharmaceutical sector reached a peak in 2011, with 68 mergers or acquisitions and a total transaction value of 8889.58 Million RMB. This process seems to differ in some respects from the previous wave of M&As in the 1990s. At that time, state-owned enterprises (SOEs) were the main driving force behind M&As, motivated by the governments’ aim to reform SOEs [5]. After the crisis, however, a bigger portion of M&As seem to have occurred between shareholding enterprises, even though the role of the government is still clearly visible, mainly through industrial policy actions.

The recent wave of M&As appears in line with the explicit policy strategy of the Chinese government to restructure the sector by allowing a major concentration of the industry and by accelerating the re-organization of production. Industrial policy, at least in the last decade, has been encouraging a group of firms to merge.

In 2006, the Chinese government [25] already launched a series of policies to encourage mergers and acquisitions in a number of high-technology and strategic emerging sectors, including pharmaceuticals. These policies generally aimed at facilitating M&A operations by simplifying the administrative and legal procedures required.

Another more recent and even more significant initiative, has been the establishment of the Good Manufacturing Practice (GMP) standard [26], that sets the basic qualification criteria required for entering the market. The criteria imply that only those firms qualified as GMP are allowed to produce in the pharmaceutical sector; in the absence of this qualification, firms can no longer operate. As a consequence, a large number of small and medium sized firms attempted to merge with firms that held a GMP license.

In addition to public policy action, the structure of the sector itself in China can be considered another distinctive feature. The Chinese pharmaceutical market is still highly fragmented. The main indexes of concentration CR3, CR5, and CR10 in the Chinese pharmaceutical market were 6.16%, 8.17%, 13.88% respectively in 2011,about 1/5 of those in the U.S. [27]. The annual cumulative revenues of the top ten listed Chinese companies were 49.16% of the total revenues of all listed companies, compared to 74.07% in the U.S. market. Moreover, there were over 13,000 Chinese pharmaceutical distributors in the sector in 2015, while the top 3 distributors only controlled 25% of the market share. This distribution concentration index is not only extremely low compared to those in the U.S., UK, France, or Australia (around 80%), but also compared to the average in South East Asian countries, including the Philippines, Malaysia and Thailand (where it is around 60%). As a consequence, many distributors in China became acquiring targets for pharmaceutical manufacturers who wanted to enter downstream activities to gain the typical advantages of vertical integration and to better control their supply chain.

Finally, consistent with the general motivations that push M&As in the pharmaceutical sector worldwide, the high R&D investment requirements is another important driver for M&As in China. More specifically, due to the fact that the efficiency of R&D investment in the pharmaceutical sector appears particularly low [28], companies have gradually shifted their strategy towards acquiring existing firms with patents, rather than engaging in new R&D projects.

The abovementioned trends capture the main specificities of the contemporary pharmaceutical sector in China and its ongoing restructuring process. The remainder of the paper addresses the debate on the effectiveness of such a restructuring.

## 3. Literature Background

For the purposes of this paper two broad streams of literature are pertinent (for a comprehensive review on M&As see among others Yaghoubi et al. [29]). On the one hand, there is the vast literature on M&As and their effects, which has been mainly developed within the fields of industrial economics (i.e., the traditional S-C-P paradigm and its evolutions), corporate business strategy and finance. According to the traditional S-C-P paradigm, M&As affect industrial performance by collectively changing the market competition and structure [30,31], intra-sectoral productivity [32], and inter-sectoral synergy [33].

On the other hand, there is the literature on industrial competitiveness and industrial performance (starting from the seminal contribution of Porter [34]), which has focused on measuring and investigating the determinants of competitiveness at the company, industry and country level.

The first stream of literature helps us to understand the conditions under which M&A operations are likely to be profitable and therefore likely to improve the performance of the sector where they take place. The second stream helps us to define an indicator of industrial performance, while a specific role for M&As has also emerged within this stream. Both streams of literature help us structure our main hypothesis and allow us to define several control variables that need to be taken into account when testing the relationship between M&As and the sector’s industrial performance.

### 3.1.The Effects of M&As at the Industry Level

Most of the existing contributions concentrate on the effects of M&As at the firm-level or at the economy-level. One of the few studies on the effects of M&A on the industry level, Andrade and Stafford [35] suggests that M&As can play two main roles: contributing to *expansion* and *contraction* of an industry. On the one hand, M&As can be regarded as a strategy to increase the capital base of the acquiring firms in order to leverage emerging growth opportunities; on the other hand, mergers appear to facilitate industry contraction, which in turn can improve efficiency. According to several scholars, such roles can change over time. For instance, Jensen [36] found that merger activities in the 1970s resulted in both excess capacity and technological improvement. The relationship between mergers and capacity utilization was negative in 1970s and 1980s, but in the 1990s the relationship became significantly positive [37]. This would suggest that M&As in the 1990s facilitated industrial expansion, whereas in the previous period they generated a contraction of the industry. What we conclude from this stream of the literature is that the expected effects of M&A can vary over time and depending upon the mainly expansionary or contractionary role they play in each phase and in each specific context.

A second important stream of research on M&As concentrates on their effects on market competition. Especially with reference to those operations that display a “merging for monopoly” pattern—a common feature of the M&As at the beginning of the 20th century—economists warned of the risks of decreasing the competition within industries, which might damage both firm-level and industry-level value creation [38]. More recent empirical studies, however, have found contrasting evidence on the relationship between market power and M&As [30,39,40]. Market power does not necessarily increase in terms of sales [30] after an M&A operation, however profitability, productive efficiency and negotiation power towards suppliers do, especially in the case of horizontal mergers [31]. In any case, a proxy of the level of market concentration must be considered to better understand the potential impact of an M&A on the performance of an industry.

### 3.2. M&As’ Effects and Industrial Performance

Coming to the specific effects of M&As on industrial performance, we start by broadly defining industrial performance as the ability of an industry to generate value added in a competitive context [41], a characteristic which is also typically considered as one dimension of industrial competitiveness. The literature on industrial competitiveness is vast and it is beyond the scope of this paper to review it (for a review of the concept of sectors’ competitiveness see among others [42]). Here we recall that in the early 1990s a number of studies already suggested that corporate strategic action, including diversification, mergers and acquisitions, investment into human resources and corporate culture determine industrial performance and competitiveness. Harrison et al. [43] in particular established a clear link between M&As and industrial performance. Concretely, there are at least three channels through which M&As can contribute to improving efficiency and competitiveness at sectoral level:

- Exploitation of operation synergies;
- Exploitation of economies of scale and scope;
- Cost reductions (particularly in R&D investment);

More recent papers recall these channels, underlying how the industrial structure and the organisation of a sector, together with technology and government policies, are important determinants of industrial competitiveness (see among others [44]).

The literature, however, on the other hand has also emphasised that not only is the industrial structure—and therefore its restructuring through M&As—important for the competitiveness of a sector, but at the same time the probability that M&A operations generate gains for the sector depends upon the structure of the industry. In other words, M&As influence the structure of a sector and its competitiveness, but the opposite is also true. This is an important aspect to be taken into account when modelling the empirical relationship between M&As and competitiveness at sectoral level: a number of confounders potentially influencing both variables need to be included in the analysis. We briefly list them below (Table 1 provides a full summary of the main control variables we include in our empirical model):

#### 3.2.1. M&As and Industrial Structure

Mergers are believed to increase market concentration [45], but at the same time industry concentration (see control variable 1.1 in Table 1) affects total acquisition gains [46]. It has been argued that in highly concentrated industries, acquiring firms capture higher wealth [45], especially if they come from other sectors [46]. Furthermore, a collusive merger, which is more likely to happen in a highly concentrated industry, is proven to achieve higher gains [45]. The extent to which such gains are translated into a higher competitiveness within the sector is debatable. However, this literature suggests that a measure of industrial concentration should be included as a control variable in our analysis.

Similarly, the distribution of firm size in a sector (see control variable 1.2 in Table 1) is one of the structural features of industrial organization, which is also regarded as one of the factors contributing to industrial competitiveness [47]. Several scholars found that the distribution of firm size within a sector determines the motivation and return of a merger (see among others [48,49,50]). In particular [51] suggest that the profitability of an M&A operation is positively associated to the ratio of the size of the largest firm to the size of other firms in the industry. We therefore include a proxy for the distribution of firm size in our empirical model.

#### 3.2.2. M&As and Macro Factors

In addition to the above-mentioned aspects, some features of the *macro*-environment are important determinants of the success of M&As [35,37,52,53].

Among these features are, for instance, changes in the international rules and regulations that can affect firms’ value and acquisition premium [54,55,56,57], as well as the growth stage of an industry in its lifecycle. The extant literature suggests that acquisitions occur in waves [35,37]. Generally, firms undertaking acquisitions during the growth phases of an industry are more likely to improve their performance. Moreover, in these stages first-movers appear to gain higher advantages and stock returns [58,59]. In growing industries M&As appear to be underlined by the search for growth and diversification [60], while in declining industries they are more likely to seek acquisitions in unrelated sectors [58,60], which in turn worsen the performance of the industry over time.

Furthermore, an industry shock (see control variable 2.1 in Table 1) can alter an industry’s structure inducing takeovers and restructuring activities through M&As [37,53]. In the case of China, important industry shocks would include the specific industrial policies targeting selected sectors, which have been proven to impact the sectors’ industrial performance [61]. Being recognized as a pillar industry or a strategic emerging sector in the Five-Year Plans for National Economic and Social Development is a clear indication of the attention of the government towards a sector and it constitutes a typical kind of industrial shock [62].

In addition, economy-wide changes such as the emergence of technologies, changes in anti-trust policies and changes in bankruptcy regulations can partly explain the changes in frequency of M&As [36].These macro factors can create opportunities for firms to improve their performance through M&As [32]. Toxvaerd [63] suggested that fundamental economic changes influence merger profitability. In the time span we considered for this analysis a fundamental change in the macro-environment that should be accounted for is, for instance, the global financial crisis of 2008. Moreover, in the last three decades, China has gradually integrated with the global market, allowing imports, inward FDI and mergers in almost all manufacturing sectors (see control variable 2.2 in Table 1). Such integration has contributed to the industrial performance and competitiveness of China [64,65]. For this reason, we also take foreign M&As targeting Chinese firms and Chinese M&As abroad into account in our analysis.

#### 3.2.3. M&As and the Characteristics of the Target and the Acquirer

Beyond the macro factors, the literature on M&As has also identified a number of characteristics of the target and acquired firm (see control variable 3.1 in Table 1) which affect the success of an M&A operation. We do not review all of them here (see among others [66,67]), however it seems clear that the more different the target and the acquirer, the more difficult the process of conducting a successful M&A. In this sense geographical, organizational and technological differences [68,69,70] are important determinants of the outcomes of M&As at the firm-level, which can translate into important effects on the competitiveness of a sector (in previous studies, the authors have found that in the particular case of China, it can be argued that the domestic market is characterized by a high fragmentation [71]. Market fragmentation means that the regional markets are very different, which is a fact that cannot be ignored when doing business across different provinces in China [72,73]. In this case, cross-province M&A in China can have different performances compared to M&A in the same province). Mainly for this reason, due to differences between the target and acquiring firms, the literature distinguishes horizontal, vertical and conglomerate mergers. The third case has long been identified as the most problematic in terms of both post-merger performance and improved competitiveness of an industry [74,75]. Vertical M&As have been considered an effective way of improving R&D capability, technological innovation and competitive advantages along the value chain [76,77,78]. In the pharmaceutical sector in particular, vertical M&As can improve control over final markets, with positive effects on both sales and margins (in the case, for instance, of the acquisition of a distributor) or can reduce input costs (in the case of upstream M&As). As concerns horizontal M&As, the debate is still open. On the one hand, thanks to scale and scope advantages, production and R&D efficiency are enhanced after an horizontal merger [79,80]; on the other, horizontal mergers are also considered to be a way to achieve monopolies and harm competition within an industry [81,82]. Especially in the case of horizontal M&As, it is crucial to understand whether the industry is experiencing an expansionary or contractionary phase (see Section 3.1). In the latter case, the risks of negative effects on the competitiveness of a sector are higher.

Finally, our focus on the specific case of China leads us to consider Chinese literature on M&As. From this literature, we understand that the intensity of M&As in other sectors can also determine the performance of a specific industry. The Chinese literature Hasid entified a variety of M&A efficiency and synergy effects among sectors [83,84]. Li and Zhang [85] empirically tested the difference in M&A trends across sectors, suggesting that when analysing M&A performance in an industry, a comparison with the trends in other sectors becomes crucial.

As for the specific effects of M&As in the pharmaceutical sector in China, scholars have found differences in the impacts on the firm and market levels. Lei and Ma [86] found that mergers among pharmaceutical firms have positive EVA one year after the merger, but negative EVA in the second year. They also found that M&As that involve a higher ownership acquisition improve the EVA. However, Qiu et al. [87] found that post-merger performance in the Chinese pharmaceutical sector is, in general, rather unstable over time.

### 3.3. Hypothesis and Model

The vast literature summarized above highlights two major motivations for M&As: one is to increase market power and the other is to decrease costs. Particularly in the pharmaceutical sector, mergers have been identified as the most important way to reduce R&D expenditure and to better organize the drug production pipeline, according to their patents’ expiration, by acquiring firms with longer lasting patents, rather than investing in new drugs discovery [15]. Globally, mergers occurred in waves in the last three decades. Most global pharmaceutical giants formed along with these waves. However, in China, there are still few domestic giants in the pharmaceutical sector and the sector appears much less concentrated than it is in other countries.

The recent trends in the pharmaceutical sector in China (Section 2), as well as recent analyses [88,89] point to three important characteristics of the industry:

- low degree of concentration;
- rapid growth;
- absence of overcapacity;

These three features allow us to hypothesize that M&As in the pharmaceuticals sector in China are being undertaken with the objective of an expansionary function in a growing sector. For this reason, we hypothesize a positive relationship between M&A intensity and industrial performance in the Chinese pharmaceutical sector, at least for horizontal and vertical M&As.

**Hypothesis** **1 (H1).** After controlling for the intensity of conglomerate M&A activity and other relevant factors, in the pharmaceutical sector an increase in the number of horizontal M&As has a positive impact on industrial performance.

**Hypothesis** **2 (H2).** After controlling for the intensity of conglomerate M&A activity and other relevant factors, in the pharmaceutical sector an increase in the number of vertical M&As has a positive impact on industrial performance.

In sum, to test these hypotheses, we set up the basic equation of the model as follows:

(1) $$y_{it}=\;\alpha_{t}+\beta_{1}x_{it}+\beta_{2}w_{it}+\beta_{3}z_{it}+\beta c_{it}+\varepsilon$$

where $y_{it}$ refers to an index of industrial performance for each industrial sector, for each year under observation (see Section 4.2), $c_{it}$ refers to a set of control variables; $x_{it}$ refers to the number of horizontal M&As in each sector and in each year of observation, $w_{it}$ refers to the number of vertical M&As; $z_{it}$ is a dummy variable that takes the value of 1 when the acquiring firm belongs to the pharmaceutical sector. ε refers to the usual error term.

## 4. Materials and Methods

### 4.1. Data

Our data are collected from three sources. First, the data about companies M&A activities were collected from Zero2IPO Database System, China, which is collated by Qingke Group, founded in 2001 and regarded as one of the most professional and authoritative research institutions in China. Its database covers almost all data on Chinese Limited Partner (LP), Venture Capital (VC), Private Equity (PE), Initial Public Offerings (IPO), M&A, Private Equity Real Estate (PERE), etc. Then we counted all the events of M&A activities at the 2-digit sector level, as defined in the *Industrial Classification and Code Standard of the National Economy of China* (Version 2011, i.e., GB/T 4754-2002).

Second, industrial-level data were collected from the “*China Industry Statistical Yearbook*”, issued by the National Bureau of Statistics, China. This series of yearbooks provide industrial data at the 2-digit according to the same classification. Third, data about the technological innovation of each sector were collected from “*China Statistical Yearbook on Science and Technology*”, issued by National Bureau of Statistics, China. This series of yearbooks also provide industrial data at the 2-digit level.

We collected the data from 2005 to 2013 for all 2-digit industrial sectors of China (including manufacturing sectors as well as the Mining and Quarrying sector and Utilities, i.e., 2-digit code from 06 to 46 according to GB/T 4754-2002 and 2011) (further, because the document of *Classification and Code Standard of National Economy Industry of China* has been adjusted twice since 2000 to present, our data are formed by two versions of *Classification and Code Standard* (version 2002 and 2011). These two versions of classification of industry are slightly different, in which some 2-digit sectors cannot be calculated directly. To unify the variety of 2-digit industrial codes between different versions, we further transferred all the code of 2-digit sectors into the version 2011 and re-collect the data). We have a total of 324 observations in our database.

### 4.2. Measuring Industrial Performance

The industrial performance of manufacturing sectors is the dependent variable in our study. In order to measure such industrial performance we develop a *composite indicator*. Composite indicators are useful tools to analyze complex phenomena, in order to “synthesize” the information provided by different variables in a single value. Composite indicators are used in particular to assess variables that are difficult to observe or measure [90,91]. For instance, composite indicators are very often used to compare country performances in terms of development, competitiveness, security, education, health, human rights, environment, corruption and financial risk [92]. These are general concepts that are difficult to measure with a single variable. Composite indicators are used to combine the information included in different variables, allowing a more precise description of the phenomenon. At the same time, as we shall see, they ensure that such a description does carry additional information, excluding inconsistency or redundancy. The aim is generally to inform policy makers, international institutions, investors and citizens about trends and changes in particular phenomena.

A composite indicator is particularly useful for our analysis, since we need to measure the trend in the *industrial performance* of sectors and this is, without a doubt, a complex phenomenon which is difficult to quantify (for a review of similar applications of composite indicators see, in particular, [42,71,93,94]). As explained in Section 2, in fact, the restructuring of the sector can only be fully understood together with the notion of *strategic* industries for the Chinese economy. The restructuring is taking place partly because the sector is considered to be a *strategic emerging* industry by the Chinese government. In this sense its performance must be related to at least four important aspects of the industry: its degree of competitiveness on external markets; its future growth prospects; its contribution to the Chinese economy; and its degree of innovativeness.

We therefore develop a composite indicator in order to classify *J* = 36 Chinese industries on the basis of their industrial performance. Our *Industrial Performance Index* (IPI) is composed by *K* = 6 variables: export sales (RMB); total assets; total profits; annual average number of employees; value added tax payable; intramural expenditure on S&T activities. These variables have been chosen to consider the industrial performance of sectors from different angles, such as: the ability of sectors to compete in international markets; to generate capital accumulation and profits (taken as a proxy of the future growth prospects); to generate employment and to produce fiscal revenues (which captures the contribution to the Chinese economy) and to invest in innovation. Statistically, a requisite for the choice of the variables is that they have an adequate degree of correlation. Variables must neither be negatively correlated nor have an excessive degree of correlation [42,71]. Indeed, a negative correlation between the variables would imply that different variables are inconsistent in describing the same phenomenon (i.e., industrial performance), distorting the analysis. At the same time, an excessively high positive correlation between two variables (e.g., above 0.80) means that one of the two is redundant in describing the phenomenon and should be deleted. In our case the chosen variables generally display a positive and moderate degree of correlation. From a conceptual point of view such positive correlations are a further support to our hypothesis that M&A are being used with an expansionary function in a growing sector, with positive effects on employment, sales and capital accumulation, in addition to profitability.

Industrial performance variables are combined using the standard methodology for building composite indicators. The procedure, is based on two steps (see, in particular, [95,96,97]):

1. normalisation
2. weighting and aggregation

In the first step of the procedure (before performing the aggregation step), the variables are normalised since they have different scales and dispersions. Let *X_jk_* denote the value of *X_k_* for sector *j*. *X_jk_* is transformed into:

(2) $$\beta \left(X_{jk}\right)=\frac{X_{jk}-min_{j}\left(X_{jk},\;j=1,\ldots ,J\right)+1/J}{max_{j}\left(X_{jk},j=1,\ldots ,J\right)-min_{j}\left(X_{jk},j=1,\ldots ,J\right)+2/J}$$

corresponding to well-known linear scaling in the min-max range. Note that, to avoid *β*(*X_jk_*) values equal to 0 or 1, which may cause computational inconsistencies in the aggregation step, correction factors 1/*J* and 2/*J* are added respectively to the numerator and denominator.

In the second step, the normalized variables are aggregated by applying an appropriate combination function. This phase implies the choice of the combination function as well as the weight to assign to each variable, in order to incorporate their different degree of importance in the indicator (for a deeper discussion on normalisation and aggregation functions, see [95,96]). In our case the variables are assigned the same weight and are combined by applying the *additive* function, obtaining for each sector *j* the values of the Industrial Performance Index (IPI), as defined by Equation (3):

(3) $$IPI_{j}=-\overset{K}{\underset{k=1}{\sum }}w_{k}\;\beta \left(X_{jk}\right)$$

where *K* is equal to 6 (number of variables); *β*(*X_jk_*) is the *X* normalized value of the *k*-th variable for the *j*-th sector; and *W_k_* is the weight given to the *k*-th variable.

**Figure 2:**
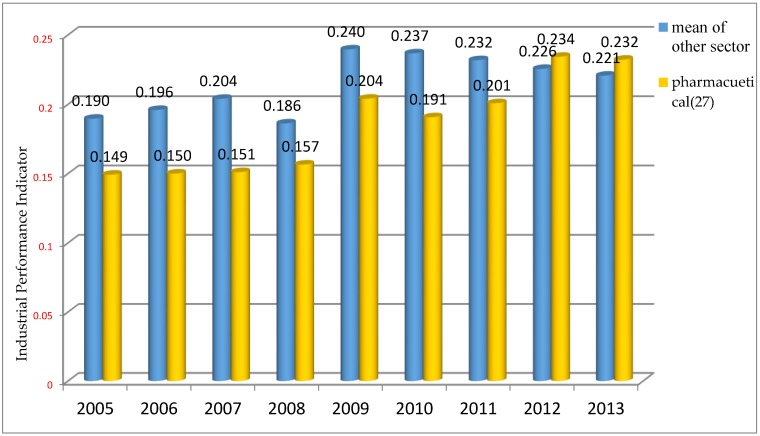
Industrial performance of pharmaceutical compared to other industrial sectors. Source: authors’ elaboration. Industrial performance indicator.

The IPI ranks the different industrial sectors, over time, according to their relative degree of performance. Industries with higher value of the IPI display higher industrial competitiveness, while those with lower values of the index are less competitive. Figure 2 shows that the pharmaceutical sector has been improving its performance over time, particularly since 2009. Indeed, since 2012 the sector’s performance has been higher than the average of other industrial sectors in China.

### 4.3. Explaining Industrial Performance

Among the factors that explain industrial performance we are particularly interested in the direction of the effects of M&As in the pharmaceutical sector. Our main explanatory variable is therefore an interaction term between the frequency of M&As and the dummy variable capturing the pharmaceutical sector. In doing so, we distinguish horizontal M&As, vertical M&As and conglomerate M&As. To this aim, we carried out in-depth studies of every M&A event based both on the description provided by Zero2IPO Database System as well as a literature review (specifically: (a) we identified the 3- and 2-digit codes of each acquirer and target; (b) we consider the event whose acquirer and target are in the same 3-digit industry as an horizontal merger; (c) vertical mergers are instead those in which the acquirer and targets have up- or down-streams relations. All other M&A are categorized as conglomerate mergers).

**Figure 3:**
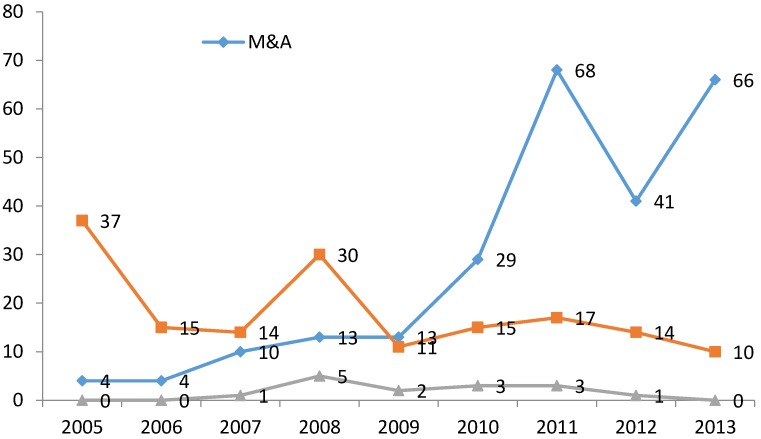
Intensity of overseas M&A and inward M&A, compared to Chinese M&A in the pharmaceutical sector. Source: authors’ elaboration.

**Figure 4:**
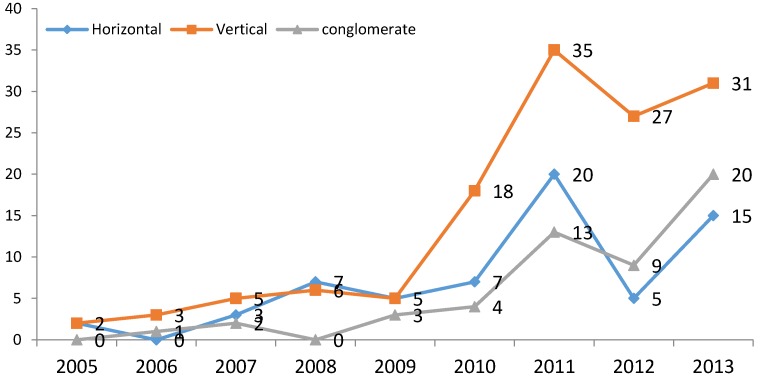
Frequency of different types of Chinese M&A in the pharmaceutical sector. Source: authors’ elaboration.

Figure 3 and Figure 4 show the general trends in M&As in the pharmaceutical sector for the period 2005–2013.

First of all, (Figure 3), a clear upward trend in M&As between Chinese firms is notable since 2009. This provides some initial evidence of a process of internal restructuring in the sector. In parallel to this trend, there is a slightly decreasing tendency in both M&As involving foreign companies and in overseas M&As by Chinese firms. Mergers between Chinese firms have therefore represented the main tool for the restructuring of the sector. Figure 4, on the other hand, shows that amongst Chinese M&As, the preferred mode is vertical M&A, followed by horizontal M&A, at least up to 2011. However, in the last two years of observations, conglomerate M&As have surpassed horizontal ones in number. Vertical M&As are largely characterized by the acquisition of distributors by manufacturing companies [98]. Overall, comparing the trends in industrial performance (Figure 2) and in M&As (Figure 4), in particular since 2009, it seems reasonable to expect some positive relationship between the accelerating restructuring process of the pharmaceutical sector and its improved industrial performance, as measured by our performance indicator.

Beyond our main variables of interest, we have collected a number of control variables, summarized in Table 1.

**Figure 5:**
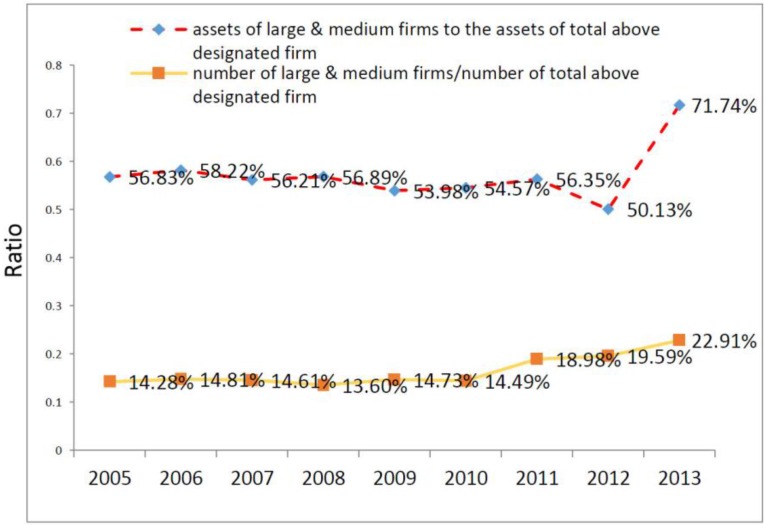
Market concentration of the Chinese pharmaceutical sector (proxied by assets of large&medium firms and number of large&medium firms over total). Source: authors’ elaboration.

Among the control variables, it is worth saying a few words on the proxies capturing market concentration. As explained in Section 3.2.3 and Section 3.3 the existing degree of concentration, is one of the key variables that impacts on the expected outcomes of (horizontal) M&As. In spite of rapid growth of M&As, according to our dataset market concentration in the Chinese pharmaceutical sector has not changed too much since 2005. Figure 5 shows a proxy for the market concentration of the Chinese pharmaceutical sector, as measured by our two main indexes (Table 2). Up to 2012, the ratio of assets of large and medium firms to total assets of all above designated size (ADS) firms was around 50% (roughly the same figure of 2005), while the ratio of the number of large and medium firms to the total number of ADS firms was less than 20% (compared to 14% of 2005). In 2013, these trends changed. The weight of large firms in terms of assets reached over 70%, while the weight of large firms in terms of number reached 22% (Figure 5). There is a visible change in the trends of the two ratios in the last year of observation. Moreover, it should be noted that in terms of assets, the market concentration of 2013 can be considered slightly high compared with the industry standard in, for instance, the U.S. and Japan. In terms of numbers, the market concentration is still low implying that numerous firms with a small amount of assets still exist in the Chinese pharmaceutical sector. The sharp increase in assets of large firms of 2012 can be attributed mainly to a significant wave of investment directed to the sector through the stock market and venture capital [99].

## 5. Results and Discussion

Table 2 presents the main results of the regression analysis; we show the pooled estimation—even though we expect this model to place excessive restrictions on our data—as well as a comparison between fixed effects and random effects estimations. Since we are interested in the specific effect of M&As in the pharmaceutical sector, the random effects (RE) model is the only one that allows us r to estimate a sector-specific beta coefficient. For a recent review of the debate on the advantages and disadvantages of FE versus RE modelling, particularly in the social sciences, see [100]. A comparison between the two models shows however, that the coefficient of our M&A-related variables are very similar in the FE and RE estimations (the pooled specification (POOL) assumes a constant intercept and slope that is equal for all sectors. The fixed effect (FE) and random effect (RE) specifications assume that there is an individual unobserved heterogeneity that produces different effects for each sector. In the fixed effect form, we assume that such unobserved individual effects are correlated with our explanatory variables, whereas they are not correlated in the random effect specification. Both estimations have advantages and drawbacks. In particular, the FE form allows us to take into account omitted time-invariant explanatory variables, and it is particularly suitable if the sample represents the entire population of interest. However, the FE model produces estimates by essentially taking into account the within variability, that is, the variation over time of each individual unit of analysis from its own mean. Therefore, variables that have a low variability over time may be incorrectly estimated. Most of our variables of interest tend to vary across individual units just as much as they do over time (or even more in many cases). RE estimates make efficient use of both within and between variability (within each unit of observation and across units). However, if the FE is the valid model, RE estimates are inconsistent). In addition, we should emphasise that since we have a composite indicator as a dependent variable, the precise interpretation of the beta coefficient is non-trivial and does not have a univocal economic interpretation. What we are interested in is mainly the *sign and the significance* of the general relationship between M&As and sectoral industrial competitiveness, rather than the precise magnitude of the coefficient. The estimations have been performed with STATA software and robust standard errors have been used in all of them. As clarified by, amongst others [101,102], when using the “robust” option in both fixed-effects and random-effects models, STATA controls for both heteroscedasticity and serial correlation. As already mentioned, the trends, both in the performance of the sector and in M&As, look significantly different before and after 2009. For this reason, we decided to also perform separate estimations for these two periods of time. The results are far more stable and the coefficients are much closer in magnitude and significance when comparing the FE and RE specifications. We present these results in Table 2.

The first results suggest a positive and significant relationship between vertical M&As and industrial performance in the pharmaceutical sector, in line with our first hypothesis. This result is however limited only to the post-2009 period of observation. At the same time, however, the relationship between horizontal M&As and sector performance seems to be negative (after summing up all the significant coefficients involving either the dummy for the pharmaceutical sector or the variable capturing the horizontal M&As). Again, this result is particularly evident after 2009 and it is contrary to our hypothesis. The other variables of the model are by and large in line with expectations. Conglomerate M&As tend to have negative effects on the sectors’ performance. In addition, the variable measuring inward M&As shows some positive effect, even though it is unstable. The variables capturing the crisis, as well as major policy shocks do not display significant effects in this formulation, although they do in the whole-period estimation (see Appendix A). As a final step, we further investigated the results related to horizontal M&As, as they are contrary to what we hypothesized. In particular, we tried to understand whether this result is somehow related to increased concentration of the sector that could have generated inefficiencies or excess market power, to the detriment of performance. We therefore introduced further interaction terms between horizontal M&As and our two measures of concentration (assets of large firms and number of large firms over total) in the pharmaceutical sector [72].

Results are shown in Table 3 and Table 4. We have also performed these estimations for the two separate periods and the results hold for the years since 2009.

The results suggest that, in the Chinese pharmaceutical sector, when horizontal M&As increase and at the same time the *assets* of large firms increase relative to the sector, the performance of the sector tends to decline (summing up all significant coefficients of the triple interaction variables). This results hold also when the ratio number of large firms is constant. On the other hand, if horizontal M&As take place together with an increase in the *number* of large firms, the performance of the sector appears to improve. 

Given that a firm normally grows in size in two ways: independent investment and M&As [35], we think that our contrasting results on the weight of large firms on the sector and horizontal M&As can provide interesting insights into the Chinese pharmaceutical industry. These findings suggest that, if existing large firms increase their assets (also through M&As) in the sector, this may eventually worsen industrial performance. On the other hand, if new large firms emerge, also through horizontal M&As, this may have a positive impact on industrial performance. It may be that at this stage in the development of the sector, it is more important to stimulate competition between large players (also new large players), than to induce existing players to increase their investment.

If these first results are confirmed by further studies, the general trend of an increasing number of large firms in the pharmaceutical sector in China that we observe in Figure 5 appears to be a good strategy for the restructuring of the sector. The results also provide support to the policies undertaken by the government in this respect. They would also be in line with a general sectoral context which is still characterized by a large number of small firms and which is experiencing an expansionary phase. At the same time, our results indicate that the peak we observe in asset concentration of large firms in 2013 and the increase in conglomerate M&As may create future problems for the performance of the sector. In Appendix A, we also report additional different model specifications, as well as basic correlation tables.

## 6. Conclusions

Ageing trends and increased income in the so-called emerging countries have effects on both the global demand of medicines and on the organization of the supply of such goods [73,103]. Studying a country like China, given its important weight both in terms of population and manufacturing capacity, is crucial to better understanding such changes. When a fast industrialising country, like China, experiences significant improvements in living standards, as well as a trend towards an ageing population, it is evident that it will stimulate a reorganisation of both the demand and supply of health-related products.

Such reorganisation is likely to trigger a restructuring in health-related sectors such as pharmaceuticals. In this paper we have offered initial evidence of such an effect. China has been promoting a process of restructuring in its pharmaceutical sector in order to become a global competitor in this field. This strategy is coherent with the long-term mission of upgrading Chinese production capacity [93,104].

In order to restructure its pharmaceutical sector, China has firstly focused strategic attention on the sector, as evidenced by the government’s Five Year Plans. In addition, it has specifically encouraged mergers and acquisitions as one of the main tools to restructure the sector and gain competitiveness. The extent to which this strategy is producing the desired effects has been largely unknown and this is a key contribution of our study.

Our main results show, firstly that there has been a large increase in the number of M&As after 2009, and particularly in vertical M&As. At the same time, we have built a composite index of industrial performance for all manufacturing sectors, including pharmaceuticals. The index shows improvements in the performance of pharmaceuticals that are, on average, higher than other manufacturing sectors for the same period. We then tested the relationship between horizontal and vertical M&As and our index of industrial performance. Our results suggest that, particularly in the post-crisis period, restructuring through vertical M&As was positively correlated with competitiveness. As for horizontal M&As, they seem to have had positive effects when they stimulated the emergence of new large firms. On the other hand, however, when both M&As and the share of assets of existing large firms increased they appear to have had a negative effect on general sectoral competitiveness. These results are coherent with what is suggested in the literature for a sector that is in an expansionary phase of its life cycle and still characterized by a low number of big players. In such a phase, a general increase in the number of big players—and possibly in the level of competition among them—can have a more positive impact on the overall performance of the sector, than higher concentration of assets in a few existing firms. As a whole, our results offer some supportive evidence for the effectiveness of the main restructuring strategies undertaken by the sector in the latest years, although some doubts emerge on the effect of the very recent wave of conglomerate M&As and growth in market assets of existing large firms.

## Figures and Tables

**Table 1:** Main control variables.

Control Variables	Measurements
*1. Industrial structure*	
1.1. Industry concentration	Number of large & medium firms/number of total above designated size firms
	Number of listed firms/number of total above designated firm
1.2. Distribution of firm size	Assets of large & medium firms to the assets of total above designated firm
	Assets of listed firms to the assets of total above designated firm
*2. Macro factors*	
2.1. Industry shocks	GMP Policy Targeting Pharmaceuticals (2010 year dummy)
2.2. Global impact	Global Financial Crisis (2009 year dummy)
	Inward FDI: foreign equity to total equity
	Number of overseas M&As by Chinese firms
	Number of M&As in China by foreign firms
*3. Characteristics of the target and the acquirer*	
3.1. Characteristics of the target and the acquirer	Total value of M&A transaction
	Percentage of the number of M&As which are cross-province

Source: authors’ elaboration.

**Table 2:** Table of results on the effects of M&A in the pharmaceutical sector (two-way interactions).

Variable	POLS	FE	RE
**After 2009**			
Horizontal_M&A	0.0025 ***	0.0019 **	0.0025 ***
Vertical_M&A	−0.0005	−0.0009	−0.0005
Conglomerate_M&A	−0.0015 *	−0.00149976 *	−0.00152168 *
Inward_FDI	−0.1670	−0.3365 *	−0.1670
N_large_firms	−0.1811 ***	−0.1956 ***	−0.1811 ***
Assets_large_firms	0.0592 **	0.0495 *	0.0592 **
Overseas_M&A	0.00001	−0.0007	0.00001
Inward_M&A	0.0022 *	0.0018	0.0022 *
Pharmaceutical	−0.0370	(omitted)	−0.0370
Pharmaceutical * Horizontal_M&A	−0.0050 ***	−0.0041 ***	−0.0050 ***
Pharmaceutical * Vertical_M&A	0.0032 ***	0.0034 ***	0.0032 ***
year2009	0.0048	0.0060	0.0048
year2010	−0.0025	−0.0025	−0.0025
Constant	0.2327 ***	0.2585 ***	0.2327 ***
N	180	180	180
**Before 2009**			
Horizontal_M&A	−0.0015	−0.0001	−0.0015
Vertical_M&A	0.0059 **	0.0044 *	0.0059 **
Conglomerate_M&A	−0.0033	−0.0039	−0.0033
Inward_FDI	0.1709	0.0639	0.1709
N_Large_firms	0.3141 **	0.1508	0.3141 **
Assets_large_firms	−0.0540 *	−0.0590 *	−0.0540 *
Overseas_M&A	0.0001	−0.0006	0.0001
Inward_M&A	0.0002	−0.0009	0.0002
Pharmaceutical	−0.0580 *	(omitted)	−0.0580 *
Pharmaceutical * Horizontal_M&A	0.0004	0.0035	0.0004
Pharmaceutical * Vertical_M&A	−0.0022	−0.0067	−0.0022
Constant	0.1684 ***	0.1915 ***	0.1684 ***
N	132	132	132

Source: authors’ elaboration. Significance: *** <1%, ** <5%, * <10%.

**Table 3:** Table of results on the effects of M&A in the pharmaceutical sector (three-way interactions—assets of large firms).

Variable	POLS	FE	RE
Horizontal_M&A	−0.0227 *	0.0046	0.0050
Vertical_M&A	0.0108 ***	0.0017	0.0022 **
Conglomerate_M&A	−0.0059	−0.0023 **	−0.0023 *
Inward_FDI	0.2518	−0.2401 *	−0.0792
Assets_large_firms	0.1549 ***	−0.0208	−0.0035
Overseas_M&A	0.0071 ***	−0.0014 *	−0.0006
Inward_M&A	0.0090 **	0.0050 **	0.0057 ***
Pharmaceutical	0.5593	(omitted)	0.3011 **
Pharmaceutical * Horizontal_M&A	−0.0662	−0.0408 ***	−0.0468 ***
Pharmaceutical * Vertical_M&A	−0.0019	0.0010	0.0011
Horizontal_M&A * Assets_large_firms	0.0493 ***	−0.0012	−0.0009
Pharmaceutical * Assets_large_firms	−1.2338	−0.5048 **	−0.5950 ***
Pharmaceutical * Horizontal_M&A * Assets_large_firms	0.0925	0.0616 ***	0.0670 ***
year2009	0.0493 *	0.0335 ***	0.0340 ***
year2010	0.0392	0.0186 **	0.0199 ***
Constant	0.0102	0.2339 ***	0.2003 ***
N	312	312	312

Source: authors’ elaboration. Significance: *** <1%, ** <5%, * <10%.

**Table 4:** Table of results on the effects of M&A in the pharmaceutical sector (three-way interactions—number of large firms).

Variable	POLS	FE	RE
Horizontal_M&A	0.0031	0.0068 ***	0.0072 ***
Vertical_M&A	0.0107 ***	0.0018 *	0.0023 **
Conglomerate_M&A	−0.0069 *	−0.0025 **	−0.0026 *
Inward_FDI	0.2735	−0.3112 **	−0.1283
N_large_firms	0.3733 ***	−0.0868 *	−0.0332
Overseas_M&A	0.0080 ***	−0.0014 *	−0.0006
Inward_M&A	0.0093 **	0.0053 ***	0.0058 ***
Pharmaceutical	−0.5021	(omitted)	−0.2882 ***
Pharmaceutical * Horizontal_M&A	−0.0174	−0.0017	−0.0029
Pharmaceutical * Vertical_M&A	−0.0069	−0.0018	−0.0019 *
Horizontal_M&A * N_large_firms	0.0249	−0.0142	−0.0137
Pharmaceutical * N_large_firms	2.5681	1.6582 ***	1.7983 ***
Pharmaceutical * Horizontal_M&A * N_large_firms	0.0217	−0.0173	−0.0171
Year2009	0.0574 **	0.0298 ***	0.0318 ***
Year2010	0.0450 *	0.0129	0.0161 *
Constant	0.0373	0.2276 ***	0.2076 ***
N	321	321	321

Source: authors’ elaboration. Significance: *** <1%, ** <5%, * <10%.

## Appendix A

**Table A1:** Descriptive statistic and table of correlations.

	Mean	S.D	1	2	3	4	5	6	7	8
1 Horizontal_M&A	1.799	3.577	1.000							
2 Vertical_M&A	3.232	7.009	0.551 **	1.000						
3 Conglomerate_M&A	1.732	4.186	0.426 **	0.612 **	1.000					
4 Inward_FDI	0.143	1.062	−0.129 *	−0.106	−0.115 *	1.000				
5 Assets_large_firms	0.517	0.198	0.258 **	0.213 **	0.199 **	−0.104	1.000			
6 Overseas_M&A	5.528	6.527	0.215 **	0.292 **	0.102	0.307 **	0.056	1.000		
7 Inward_M&A	1.840	2.732	0.277 **	0.401 **	0.216 **	−0.037	0.160 **	0.494 **	1.000	
8 Pharmaceutical	0.028	0.165	0.212 **	0.228 **	0.153 **	−0.014	0.067	0.248 **	0.025	1.000

Note: * *p* < 0.05, ** *p* < 0.01, *** *p* < 0.001.

**Table A2:** Estimation of the effects of horizontal and vertical M&A on the industrial performance for the pharmaceutical sector. Period 2005–2013.

Variable	POLS	FE	RE
Horizontal_M&A	0.0073 **	0.0039 ***	0.0044 ***
Vertical_M&A	0.0108 ***	0.0016	0.0020 *
Conglomerate_M&A	−0.0066 *	−0.0019 *	−0.0019 *
Inward_FDI	0.31371205	−0.3000 **	−0.1412
N_large_firms	0.2521	−0.0880	−0.0465
Assets_large_firms	0.1382 **	−0.0088	−0.0007
Overseas_M&A	0.0073 ***	−0.0015 **	−0.0009
Inward_M&A	0.0094 **	0.0050 **	0.0055 ***
Pharmaceutical	−0.1544 ***	(omitted)	−0.0350
Pharmaceutical * Horizontal_M&A	−0.0163 ***	−0.0061 ***	−0.0072 ***
Pharmaceutical * Vertical_M&A	0.0002	0.0018	0.0020 *
Year 2009	0.0537 **	0.0313 ***	0.0330 ***
Year 2010	0.0387	0.0150 *	0.0177 **
Constant	−0.0207	0.2372 ***	0.2130 ***
N	312	312	312

Note: * *p* < 0.05, ** *p* < 0.01, *** *p* < 0.001.

## References

[1] Peng D., Yang H. (2010). China’s population ageing and active ageing. China J. Soc. Work.

[2] Rubini L., Pollio C., Di Tommaso M.R. (2017). Transnational Research Networks in Chinese Scientific Production. An Investigation on Health-Industry Related Sectors. Int. J. Environ. Res. Public Health.

[3] Comanor W.S. (1965). Research and technical change in the pharmaceutical industry. Rev. Econ. Stat..

[4] Zeng J. (2014). On development briefing and trend prediction of Chinese pharmaceutical sector. Rev. Econ. Res..

[5] Yin H., Salmon J.W. (2003). Strategic restructuring of China’s pharmaceutical industry: Mergers and acquisitions. J. Pharm. Mark. Manag..

[6] Thompson D.E. (2001). Get big enough (but not too big) to source innovation. Res. Manag..

[7] Wang F. (2015). On the mechanism of innovative network transferring in biochamical pharmaceutical industry: Case of Zhangjiang. Sci. Res. Manag..

[8] Cheri G. (2004). The Effect of Changing Intellectual Property on Pharmaceutical Industry Prospects in India and China.

[9] Hu Z. (2007). A Study on the Equilibrium Exchange Rate and Dislocation Degree of RMB Behavior. World Economy Study.

[10] National Bureau of Statistics China Industrial Statistical Yearbook. http://www.stats.gov.cn/tjsj/ndsj/2013/indexeh.htm.

[11] The State Council of the People’s Republic of China (SCC) (2011). 12th Five Year Plan of China’s National Strategic Emerging Industry Development Planning.

[12] Cockburn I., Henderson R. (2001). Scale and scope in drug development: Unpacking the advantages of size in pharmaceutical research. J. Health Econ..

[13] Lan H. (2012). From Champion of China to Champion of World.

[14] Hu X., Wu X. (2015). Effects of M&A strategy on performance of Chinese pharmarceutical firms: Empirical research with Chinese public pharmaceutical corporates. Forum World Econ. Politics.

[15] Grabowski H., Kyle M., Gugler K., Yurtoglu B.B. (2008). Mergers and alliances in pharmaceuticals: Effects on innovation and R&D productivity. The Economics of Corporate Governance and Mergers.

[16] Danzon P.M., Epstein A., Nicholson S. (2007). Mergers and acquisitions in the pharmaceutical and biotech industries. Manag. Decis. Econ..

[17] Guan C., Duan J. (2009). Empirical Study on M&A Performance of Public Pharmaceutical Companies of China. China Pharm..

[18] He X., Niu X. (2009). Analysis on M&A Performance of Chinese Public pharmaceutical companies. Econ. Res. Guid..

[19] Koenig M., Mezick E. (2004). Impact of mergers & acquisitions on research productivity within the pharmaceutical industry. Scientometrics.

[20] Bottazzi G., Dosi G., Lippi M., Pammolli F. (2001). Innovation and corporate growth in the evolution of the drug industry. Int. J. Ind. Organ..

[21] Berndt E. (2001). The U.S. pharmaceutical industry: Why major growth in times of cost containment?. Health Aff..

[22] Wang Y. (2004). The Reasons, characteristics and trends of the reorganization of China’s pharmaceutical industry. Econ. Theory Econ. Manag..

[23] Li B. (2008). Review on the research on M&A in the pharmaceutical sector. Reform. Strateg..

[24] Yin H. (2001). Merger and acquisition activity in the Chinese pharmaceutical industry. J. Manag. Care Pharm..

[25] The State Council of the People’s Republic of China (SCC) (2006). The Eleventh Five Year Plan for National Economic and Social Development of People’s Republic of China.

[26] China Food and Drug Administration (2011). Regulation on Quality Management of Medicines Production (Adjustment 2010).

[27] Liu Q., Liu F. (2014). The development of China’s pharmaceutical industry and the industrial policy’s current situation, problems and policy suggestions. Rev. Econ. Res..

[28] Zhang Y., Liu Q., Xu Y.R. (2011). Efficiency and influencing factors of China’s pharmaceutical manufacturing industry. Chin. Technol. BBS.

[29] Yaghoubi R., Yaghoubi M., Locke S., Gibb J. (2016). Mergers and acquisitions: A review (part 2). Stud. Econ. Financ..

[30] Mueller D.C. (1985). Mergers and market share. Rev. Econ. Stat..

[31] Gugler K., Mueller D.C., Yurtoglu B.B., Zulehner C. (2003). The effects of mergers: An international comparison. Int. J. Ind. Organ..

[32] Holmstrom B., Kaplan S.N. (2001). Corporate Governance and Merger Activity in the U.S.: Making Sense of the 1980s and 1990s. J. Econ. Perspect..

[33] Wang S. (2012). On M&A’s industry consolidation function and its performance. Int. Bus..

[34] Porter M. (1990). The competitive advantage of nations. Harv. Bus. Rev..

[35] Andrade G., Stafford E. (2004). Investigating the economic role of mergers. J. Corp. Financ..

[36] Jensen M. (1993). The modern industrial revolution, exit, and the failure of internal control systems. J. Financ..

[37] Mitchell M., Mulherin J. (1996). The impact of industry shocks on takeover and restructuring activity. J. Financ. Econ..

[38] Stigler G. (1950). Monopoly and oligopoly by merger. Am. Econ. Rev..

[39] Kim E., Singal V. (1993). Mergers and market power: Evidence from the airline industry. Am. Econ. Rev..

[40] Sapienza P. (2002). The effects of banking mergers on loan contracts. J. Financ..

[41] Bhattacharjea A. (2006). Labour market regulation and industrial performance in India: A critical review of the empirical evidence. Indian J. Labour Econ..

[42] Di Tommaso M.R., Tassinari M., Bonnini S., Marozzi M. (2017). Industrial policy and manufacturing targeting in the U.S.: New methodological tools for strategic policy-making. Int. Rev. Appl. Econ..

[43] Harrison J., Hitt M., Hoskisson R. (1991). Synergies and post-acquisition performance: Differences versus similarities in resource allocations. J. Manag..

[44] Bhawsar P., Chattopadhyay U. (2015). Evaluation of Cluster Competitiveness: Review, Framework and the Methodology. Compet. Forum.

[45] Eckbo B. (1986). Mergers and the market for corporate control: The Canadian evidence. Can. J. Econ..

[46] Shahrur H. (2005). Industry structure and horizontal takeovers: Analysis of wealth effects on rivals, suppliers, and corporate customers. J. Financ. Econ..

[47] Porter M.E. (1985). Competitive Advantage: Creating and Sustaining Superior Performance.

[48] Morck R., Shleifer A., Vishny R. (1990). Do managerial objectives drive bad acquisitions?. J. Financ..

[49] Lang L., Stulz R., Walkling R. (1991). A test of the free cash flow hypothesis: The case of bidder returns. J. Financ. Econ..

[50] Servaes H. (1991). Tobin’s Q and the Gains from Takeovers. J. Financ..

[51] Gorton G., Kahl M., Rosen R. (2009). Eat or be eaten: A theory of mergers and firm size. J. Financ..

[52] Mulherin J., Boone A. (2000). Comparing acquisitions and divestitures. J. Corp. Financ..

[53] Andrade G., Mitchell M., Stafford E. (2001). New Evidence and Perspectives on Mergers. J. Econ. Perspect..

[54] LaPorta R., Lopez-de-Silanes F. (2008). The economic consequences of legal origins. J. Econ..

[55] Porta R., Lopez-de-Silanes F. (1999). Corporate ownership around the world. J. Financ..

[56] Porta R., Lopez-de-Silanes F., Shleifer A. (2002). Investor protection and corporate valuation. J. Financ..

[57] Palia D. (1993). The managerial, regulatory, and financial determinants of bank merger premiums. J. Ind. Econ..

[58] Stimpert J., Duhaime I. (1997). Seeing the big picture: The influence of industry, diversification, and business strategy on performance. Acad. Manag. J..

[59] Carow K., Heron R., Saxton T. (2004). Do early birds get the returns? An empirical investigation of early-mover advantages in acquisitions. Strateg. Manag. J..

[60] Anand J., Singh H. (1997). Asset redeployment, acquisitions and corporate strategy in declining industries. Strateg. Manag. J..

[61] Jiang F., Li X. (2010). Direct intervention in the market and restrictive competition: The orientation and fundamental defect of China’s industrial policy. Chin. Ind. Econ..

[62] Meng Q., Yin X., Bai J. (2016). Does industrial policy support inspire innovation?—A natural experiment based on the “Five-Year Plan”change. South China Econ..

[63] Toxvaerd F. (2008). Strategic merger waves: A theory of musical chairs. J. Econ. Theory.

[64] Mao R. (2006). Analysis of China’s manufacturing trade competitiveness and its determinants. Manag. World.

[65] Liu L., Li W., Zhang Y. (2009). Comparative advantage, FDI and the national industries’ international competitiveness—the vulnerability analysis of international competitiveness of “Made in China”. Chin. Ind. Econ..

[66] James C., Wier P. (1987). Returns to acquirers and competition in the acquisition market: The case of banking. J. Political Econ..

[67] DeLong G.L. (2001). Stockholder gains from focusing versus diversifying bank mergers. J. Financ. Econ..

[68] Moeller S., Schlingemann F. (2005). Global diversification and bidder gains: A comparison between cross-border and domestic acquisitions. J. Bank. Financ..

[69] Conn R., Cosh A., Guest P. (2005). The impact on UK acquirers of domestic, cross-border, public and private acquisitions. J. Bus..

[70] Kiymaz H., Mukherjee T. (2000). The Impact of country diversification on wealth effects in cross-border mergers. Financ. Rev..

[71] Young A. (2000). The razor’s edge: Distortions and incremental reform in the People’s Republic of China. Q. J. Econ..

[72] Lan H. (2015). Research on Growth and Restructring of Chinese Business Groups.

[73] Huang M., Zhang H., Angelino A. (2017). Chinese pharmaceuticals: Does sub-national marketization matter? Evidence of cross-province acquisitions by Guangdong firms. Int. J. Healthcare Technol. Manag..

[74] Edwards C. (1955). Conglomerate bigness as a source of power. Business Concentration and Price Policy.

[75] Montgomery C. (1985). Product-market diversification and market power. Acad. Manag. J..

[76] Milliou C. (2004). Vertical integration and R&D information flow: Is there a need for “firewalls”?. Int. J. Ind. Organ..

[77] Buehler S., Schmutzler A. (2008). Intimidating competitors—Endogenous vertical integration and downstream investment in successive oligopoly. Int. J. Ind. Organ..

[78] Harrigan K. (1985). Exit barriers and vertical integration. Acad. Manag. J..

[79] Cohen W., Levin R. (1989). Empirical studies of innovation and market structure. Handb. Ind. Organ..

[80] Röller L., Stennek J., Verboven F. (2000). Efficiency Gains from Mergers.

[81] Aktas N., Bodt E., Roll R. (2007). Is European M&A regulation protectionist?. Econ. J..

[82] Cassiman B., Colombo M.G., Garrone P., Veugelers R. (2005). The impact of M&A on the R&D process: An empirical analysis of the role of technological- and market-relatedness. Res. Policy.

[83] Tian M. (2009). Comparison of merger efficiency based on different economic regions and industries. Sci. Financ. Econ..

[84] Zhang R., Feng J. (2007). Empirical research on mergers and acquisitions of public Chinese companies. Res. Econ. Manag..

[85] Li J., Zhang Q. (2012). Research on the difference of M&A of public companies in China from the perspective of the whole industry—Based on the method of functional data analysis. Explor. Econ. Probl..

[86] Lei Y., Ma C. (2005). Economic Value Added (EVA) and enterprise value assessment. Mod. Manag. Sci..

[87] Qiu L., Bian Y., Wang Y. (2006). Empirical study on M&A performance of public companies in China’s pharmaceutical Industry. Chin. Pharm..

[88] CITIC Futures (2012). Industrial Report about Chinese Pharmaceutical Sector.

[89] Research Team on Policy Research about Overcapacity Reduction in Research Center on Development of State Council (2015). The feature, risk and policy study of the overcapacity in China recently—Analyses based on field investigation and micro data. Manag. World.

[90] Fayers P., Hand D. (2002). Causal variables, indicator variables and measurement scales: An example from quality of life. J. R. Stat. Soc..

[91] Marozzi M. (2009). A composite indicator dimension reduction procedure with application to university student satisfaction. Stat. Neerlandica.

[92] Nardo M., Saisana M., Saltelli A., Tarantola S., Hoffman A. (2005). Handbook on Constructing Composite Indicators.

[93] Barbieri E., Tassinari M. (2017). China’s strategic sectors: Trends in health-related manufacturing. Int. J. Healthc. Technol. Manag..

[94] Tassinari M., Di Tommaso M.R. Open Innovation Practices: Measuring, Economic Performance, and Industrial Policy Issues. An Analysis of the Italian Manufacturing System. http://193.205.129.80/repec/cme/wpaper/cmetwp_06_2014.pdf.

[95] Arboretti G., Bonnini S., Salmaso L. (2007). A performance indicator for multivariate data. Quad. Stat..

[96] Bonnini S., Corain L., Cordellina A., Crestana R., Bini M., Monari P., Piccolo D., Salmaso L. (2009). A novel global performance score with an application to the evaluation of new detergents. Statistical Methods for the Evaluation of Educational Services and Quality of Products, Contribution to Statistics.

[97] Marozzi M. (2015). Measuring trust in European public institutions. Soc. Indic. Res..

[98] Xie J. (2014). Current situation of M&A in Chinese pharmaceutical sector. Times Financ..

[99] Zero2IPO (2017). Research Report on Investment in Chinese Pharmaceutical Sector.

[100] Bell A., Jones K. (2015). Explaining fixed effects: Random effects modeling of time-series cross-sectional and panel data. Polit. Sci. Res. Methods.

[101] Stock J.H., Watson M.W. (2008). Heteroskedasticity—Robust standard errors for fixed effects panel data regression. Econometrica.

[102] Stata Press (2011). Stata Longitudinal Data/Panel Data Reference Manual—Release 12.

[103] Angelino A., Khanh D.T., An Ha N., Pham T. (2017). Pharmaceutical Industry in Vietnam: Sluggish Sector in a Growing Market. Int. J. Environ. Res. Public Health.

[104] Di Tommaso M.R., Rubini L., Barbieri E. (2013). Southern China. Industry, Development and Industrial Policy.

